# Maternal Thyroid Dysfunction and Neonatal Thyroid Problems

**DOI:** 10.1155/2013/987843

**Published:** 2013-04-30

**Authors:** Hulya Ozdemir, Ipek Akman, Senay Coskun, Utku Demirel, Serap Turan, Abdullah Bereket, Hulya Bilgen, Eren Ozek

**Affiliations:** ^1^Department of Pediatrics, Division of Neonatology, Marmara University School of Medicine, Istanbul, Turkey; ^2^Yakacik Maternity and Children State Hospital, Istanbul, Turkey; ^3^Department of Pediatrics, Division of Endocrinology, Marmara University School of Medicine, Istanbul, Turkey

## Abstract

*Aim.* To investigate obstetric features of pregnant women with thyroid disorders and thyroid function tests of their newborn infants. *Methods.* Women with hypothyroidism and having anti-thyroglobulin (ATG) and anti-thyroid peroxidase (anti-TPO) antibodies were assigned as group I, women with hypothyroidism who did not have autoantibodies were assigned as group II, and women without thyroid problems were assigned as group III. *Results.* Pregnant women with autoimmune hypothyroidism (group I) had more preterm delivery and their babies needed more frequent neonatal intensive care unit (NICU) admission. In group I, one infant was diagnosed with compensated hypothyroidism and one infant had transient hyperthyrotropinemia. Five infants (23.8%) in group II had thyroid-stimulating hormone (TSH) levels >20 mIU/mL. Only two of them had TSH level >7 mIU/L at the 3rd postnatal week, and all had normal free T4 (FT4). Median maternal TSH level of these five infants with TSH >20 mIU/mL was 6.6 mIU/mL. In group III, six infants (6.5%) had TSH levels above >20 mIU/mL at the 1st postnatal week. *Conclusion.* Infants of mothers with thyroid problems are more likely to have elevated TSH and higher recall rate on neonatal thyroid screening. Women with thyroid disorders and their newborn infants should be followed closely for both obstetrical problems and for thyroid dysfunction.

## 1. Introduction

Hypothyroidism, both overt and subclinical, is common in women of reproductive age and during pregnancy, with frequencies ranging from 0.3% to 2.5% [1]. Hypothyroidism has adverse effects on the course of pregnancy and development of the fetus [2]. Several studies have reported that maternal hypothyroidism is associated with increased risks of abortions, stillbirths, preterm delivery, and pregnancy-induced hypertension [3–6]. Conversely, other reports have shown successful pregnancy outcomes in women who were profoundly hypothyroid [1]. More recently, the potential adverse impact of maternal hypothyroidism and hypothyroxinemia, even when subclinical, on neurodevelopmental outcomes in the offspring has been recognized [7–9]. Hypothyroidism should be corrected before initiation of pregnancy, replacement dosage should be augmented early in pregnancy, and euthyroidism should be maintained throughout [10]. Maternal hyperthyroidism during pregnancy is associated with an increased risk of low birth weight, predisposing to neonatal morbidity and mortality [11]. In addition, Medici et al. [12] have reported that maternal high-normal FT4 levels in early pregnancy are associated with lower birth weight and an increased risk of small for gestational age (SGA) newborns.

Thyroid-stimulating hormone surges soon after birth, resulting in thyroxine (T4) concentrations that are higher in the first postnatal week than at any other time of life and in circulating triiodothyronine (T3) concentrations that are three to four times higher than fetus. Thyroid hormone synthesis is critically dependent on an adequate prenatal and postnatal supply of iodine, which can paradoxically suppress T4 secretion when present in excess, especially in preterm infants and in the presence of iodine deficiency [13]. Congenital hypothyroidism is the most frequent cause of preventable mental retardation. Neonatal hypothyroidism has an incidence of one in 3.000–4.000 births and includes both permanent and transient types [14]. Primary congenital hypothyroidism consists of disorders of thyroid development or of thyroid hormone synthesis [15–17]. Transient congenital hypothyroidism can be due to iodine deficiency or excess, maternal consumption of goitrogens or antithyroid medications during pregnancy, transplacental passage of TSH receptor-blocking antibodies, and neonatal very low birth weight and prematurity [16]. Even transient hypothyroidism can cause adverse neurologic outcome in a newborn. Thus, early diagnosis and treatment is recommended.

The aim of this study is to investigate obstetric features of pregnant women with thyroid disorders and postnatal clinical course and thyroid function tests of their newborn infants.

## 2. Methods

The study group consisted of the pregnant women with thyroid disorders followed in the obstetric outpatient clinic at Marmara Unıversity Hospital in Turkey, in 2008–2010. The control group was composed of healthy pregnant women who presented to the obstetric outpatient clinic on the same day of the enrollment of a hypothyroid patient. Three control mothers were enrolled for each mother with thyroid disorder. The definition of maternal hypothyroidism was based on the diagnosis of an endocrinologist, and thyroid hormone replacement was done throughout pregnancy. During clinical followup, thyroid function tests including FT4 and TSH and thyroid autoantibody titers (anti-TPO and ATG) were measured. The obstetric and clinical features of patients were reviewed. Hypothyroid women with positive ATG and anti-TPO titers were assigned as group I (*n* = 13), and those who did not have autoantibodies were assigned as group II (*n* = 21), while women without thyroid problems were assigned as group III (*n* = 92). Demographic characteristics of the infants were recorded. Thyroid tests of the infants were measured in the first postnatal week and third postnatal week in the study group. Thyroid function test of the infants of control group was checked before discharge from the hospital. Serum TSH, FT4, anti-TPO, and ATG was measured by chemiluminescence assay (Roche, Switzerland). Newborns with serum TSH ≥20 mIU/L were considered abnormal at first postnatal week and were recalled for further evaluation. On the 3rd week, if TSH >7 mIU/L and FT4 <1 ng/dL, the infant was considered to have congenital hypothyroidism, and those with high TSH at the first week but normal serum TSH and T4 values at the third week were considered to have transient hyperthyrotropinemia (THT). ATG >100 IU/mL and anti-TPO >30 IU/mL were considered to be abnormal.

Informed consent was obtained from all patients, and the study was approved by institutional review board.

### 2.1. Statistical Analysis

*Mann-Whitney U* and *Kruskal-Wallis* tests were used for quantitative variables and *Fischer's* exact test was used for categorical variables. Correlation between neonatal and mother anti-TPO and ATG was assessed by Spearman's rank correlation test. *P* < 0.05 was considered significant. SPSS 17.0 was used for statistical analysis.

## 3. Results

Demographic characteristics of the patients were given in Table 1. Maternal age, parity, and previous abortus history were not significantly different among the groups. However, pregnant women with autoimmune hypothyroidism (group I) had more preterm delivery, and their babies needed more frequent NICU admission. NICU admission diagnoses were mainly respiratory distress or suspected sepsis. Rates for gestational diabetes mellitus (21.6%) and pregnancy-induced hypertension (14.5%) tended to be higher in mothers with thyroid problem (groups I-II) compared to the control group (1.8% and 2.1%, resp.). Treatment for infertility was required in 38.% (*n* = 5) of mothers in group I, 9.5% (*n* = 2) in group II, 3.2% (*n* = 3) in group III. However, due to inadequate sample size, statistical analysis was not possible. 

Six infants in group I (40%) had positive anti-TPO titers at the end of the 1st postnatal week; all except one had undetectable titers at the end of the 3rd week. Mean maternal TPO titers were significantly higher in infants with positive TPO titers compared to infants with negative titers (362.6 ± 115 IU/mL versus 47.8 ± 7.4 IU/mL; *P* = 0.001, *r* = 0,756). Ten mothers had high ATG titers whereas none of their infants had high ATG titers. In group I, one infant was diagnosed with compensated hypothyroidism. Thyroid hormone replacement was prescribed but not given to the infant by the parents. In the followup, thyroid functions tests were entirely normal at the 8th postnatal week. 

Five infants (23.8%) in group II had TSH levels >20 mIU/mL. Only two of them had TSH level >7 mIU/L at the 3rd postnatal week. During followup, all TSH values returned to normal ranges at the 8th postnatal week (Table 2). Median maternal TSH level of these five infants with TSH >20 mIU/mL was 6.6 mIU/mL. In group III, six infants (6.5%) had TSH levels above >20 mIU/mL at the 1st postnatal week, but TSH levels returned to normal at the 3rd week in all. None of the patients required treatment in group III. The results indicated a higher neonatal TSH recall rate in infants of mother with thyroid problems (*P* = 0.02). The comparison of maternal TSH and FT4 levels at 3rd trimester revealed statistically significant difference between groups indicating inadequately controlled patients among groups I and II (Table 1).

## 4. Discussion

In this study, we found that pregnant women with autoimmune hypothyroidism had more preterm delivery and their babies needed more frequent NICU admission. The infants of hypothyroid mothers had higher recall rate in newborn TSH screening and transient thyroid dysfunction in the first 8 weeks of life.

Thyroid disease is common in women of reproductive age. The frequency of thyroid deficiency varies among pregnant women in different countries and ranges between 0.19% in Japan and as high as 2.2% in Belgium and 2.5% in the United States [18–20]. Maternal thyroid deficiency, even subclinical, has been reported to be associated with adverse pregnancy outcomes that may be improved by T4 replacement [4]. Fluctuations that occur in T4 metabolism during pregnancy make it difficult to maintain meticulous normal thyroid hormone values during gestation in hypothyroid mothers [21]. Pregnancy causes increased thyroid gland vascularity, increased renal iodide clearance, and iodide losses to the foetus [1]. Prenatal vitamin supplements commonly taken during pregnancy are rich in iron and calcium, both of which inhibit thyroxine absorption [22, 23]. Many prenatal vitamins do not contain the recommended 200 *μ*g of iodine for pregnancy [24]. Fluctuations in thyroxine metabolism that occur during pregnancy may further impair maternal-foetal transfer of thyroxine despite apparently optimal maternal thyroid status [25]. Reduced foetal thyroxine may cause disruption to the development of the pituitary-thyroid axis of the newborn, foetal pituitary GH secretion, vascular responsiveness and maturation, and cardiovascular homeostasis in utero [25–27]. These factors may be responsible for the observation of a reduced neonatal birthweight of offspring born to mothers with inadequately controlled thyroid status at initial presentation and at the third trimester. Pregnant women who at first presentation had above 98 percentile of TSH levels or those whose TSH remained suboptimal in the final trimester of pregnancy may be more likely to give birth to a low-birthweight infant [1].

Several studies have reported that maternal hypothyroidism is associated with increased risks of abortions, stillbirths, preterm delivery, and pregnancy-induced hypertension [3–6]. Autoimmune thyroid disease is common in pregnancy. Subclinical hypothyroidism has been associated with miscarriage in both first and second trimesters [28]. Similarly, the presence of antibodies to thyroid peroxidase or thyroglobulin is associated with a significant increment in miscarriages [2]. In our study, 26.7% of mothers in group I had a previous abortus history, but probably due to inadequate sample size, this is not significantly different than the control group. Glinoer et al. [29] documented an increased rate of preterm birth in 87 women with autoimmune thyroid disease. In our study, preterm birth rate was increased in group I as well. Rates for gestational diabetes mellitus, pregnancy-induced hypertension, and treatment of infertility tended to be higher in mothers with both autoimmune (group I) and nonautoimmune thyroid diseases (group II) compared to the control group. However, due to inadequate sample size, statistical analysis was not possible.

Autoimmune thyroid disease inpregnancy possesses important risk factors both for the mother, the fetus, and newborn infant. The clinical and endocrinological pictures of the thyroid disease in pregnant women and their offsprings can vary greatly and mainly depend on the type and amount of the anti-thyroid autoantibodies which cross the placenta [30]. The reported prevalence of thyroid autoantibodies in pregnant women ranges from 5.2% in Belgium to 12.5% in North America. Although a uniform correlation between maternal or newborn serum thyroid autoantibodies and sporadic congenital hypothyroidism is lacking, there are many reports relating maternal autoimmune thyroid disease to transient congenital hypothyroidism in newborn thyroid screening programs [31–33]. Dussault and Fisher [34] documented that elevated TSH concentrations were more frequent (7.0% versus 0.9%; *P* < 0.001) in the mothers of hypothyroid newborns. They also documented that the prevalence of newborn transient hypothyroidism was significantly higher (27% versus 15%, *P* = 0.04) in the mothers with autoimmune thyroid disease. Korkmaz et al. [35] demonstrated that none of the newborn infants with maternal Hashimato disease in the early neonatal period had abnormal thyroid function tests or physical examination findings. In our study, six infants in group I (40%) had positive anti-TPO titers at the end of the first postnatal week, all except one who had undetectable titers at the end of the third week. Mean maternal anti-TPO titers were significantly higher in infants with positive titers compared to infants with negative titers. None of the infants of mothers with elevated Anti-TG antibodies had elevated serum anti-TG levels. These results suggest that maternal TPO levels especially the high titers are transferred to the infant and are clinically more relevant. In group I, one infant was diagnosed with compensated hypothyroidism. In our study, the results indicated a higher recall rate in newborn screening in infants of mother with thyroid problems although most of them returned to normal on followup. The median TSH levels of mothers of the infants with hyperthyrotropinemia were also high indicating inadequate control of thyroid status during pregnancy. In group I, the median TSH level of mothers was 5 mIU/L which was higher than the recommended goal (2.5 mIU/L); undertreated hypothyroidism might had an additional negative effect on developing fetus. 

Women with thyroid disorders should be followed closely throughout pregnancy for the prevention of obstetric complications, and their newborn infants should be followed closely in the first months of postnatal life for thyroid dysfunction.

## Figures and Tables

**Table 1:** Demographic and laboratory characteristics of patients.

	Group I (*n* = 13)	Group II (*n* = 21)	Group III (*n* = 92)	*P*
Maternal age (median; min–max)	31 (25–39)	29.5 (29–39)	31 (29–44)	0.6
Parity (primipar/multipar)	3/10	7/14	37/55	0.4
Previous abortus history *n* (%)	4 (26.7)	1 (4.5)	11 (12)	0.2
Previous preterm birth *n* (%)	3 (20)	0	0	*
Current preterm delivery *n* (%)	9 (60)	6 (27.3)	17 (18.5)	0.002
Multiple pregnancy *n* (%)	4 (26.7)	2 (9.1)	13 (14.1)	0.3
Maternal TSH** (median; min–max)	5 (0.9–14)	2.7 (0.2–18.9)	1.8 (1.1–3.1)	<0.001
Maternal FT4** (median; min–max)	1.1 (0.7–1.7)	0.9 (0.1–1.6)	1.2 (1–1.7)	<0.001
Birth weight (gram) (median; min–max)	2840 (970–3600)	3180 (1700–4230)	3140 (1200–4550)	0.3
Gestational age (week) (median; min–max)	36.5 (29–38)	38 (32–40)	38 (29.5–41)	0.002
Sex (female/male) (%)	33.3/66.7	40.9/59.1	54.3/45.6	0.1
Intrauterine growth retardation *n* (%)	2 (15.4)	1 (4.8)	8 (8.8)	0.6
NICU admission *n* (%)	6 (46.7)	8 (31.8)	3 (3.3)	<0.001

*Statistical analysis inapplicable.

**Measurements were obtained at 3rd trimester.

**Table 2:** Neonatal thyroid disfunction in the study group.

Patient no.	Group	Gestational age (week)	Maternal TSH level (mIU/mL)*	Maternal FT4 level (mIU/mL)*	Treatment of the mother	TSH level (mIU/mL) 1st week	FT4 level (ng/dL) 1st week	TSH level (mIU/mL) 3rd week	FT4 levels (ng/dL) 3rd week	TSH level (mIU/mL) 8th week	FT4 levels (ng/dL) 8th week	Diagnosis	Treatment
1	I	38	5.6	1.1	L-thyroxine	6.3	1.55	7.44	1.2	1.4	1.3	Transient hyperthyrotropinemia	(−)
2	I	*36 *	5.7	1.7	L-thyroxine	10.7	1.2	14.4	1.06	4.2	1.5	Transient compensated hypothyroidism	(+)
3	II	40	7.2	1.7	L-thyroxine	30.7	1.9	12.4	2.2	3.2	1.7	Transient hyperthyrotropinemia	(−)
4	II	34	0.9	1.6	L-thyroxine	21.8	2.7	3.3	1.4	—	—	Transient hyperthyrotropinemia	(−)
5	II	36	1.6	0.8	L-thyroxine	21.7	1.9	4.4	1.6	—	—	Transient hyperthyrotropinemia	(−)
6	II	38	5.3	1.2	L-thyroxine	29.7	2.2	9.9	1.6	5.8	1.2	Transient hyperthyrotropinemia	(−)
7	II	37	3.2	0.7	L-thyroxine	20.9	2.1	5.2	1.3	—	—	Transient hyperthyrotropinemia	(−)

*If TSH > 7 mIU/mL, at the third week, TSH level is rechecked every two weeks until it comes down to normal values.
